# Antioxidant activity and mineral content of *Impatiens tinctoria* A. Rich (Ensosila) tuber, an Ethiopian medicinal plant

**DOI:** 10.1038/s41598-023-41824-9

**Published:** 2023-09-11

**Authors:** Gizachew Haile Gidamo

**Affiliations:** 1 Biotechnology and Bioprocess Centre of Excellence, Addis Ababa Science and Technology University, Addis Ababa, Ethiopia; 2 Department of Biotechnology, College of Natural and Applied Sciences, Addis Ababa Science and Technology University, P.O. BOX 16417, Addis Ababa, Ethiopia

**Keywords:** Chemical biology, Natural products

## Abstract

Recently, an interest has surged in incorporating extracts of medicinal plants as active ingredients in cosmetic formulations, highlighting the need to analyze medicinal plants of cosmetic interest for phytochemicals and bioactivities. The tuber of the Ethiopian medicinal plant *Impatiens tinctoria* A. Rich (Ensosila) is used to make traditional cosmetics. The aim of the study was to investigate the antioxidant and mineral content of the *Impatiens tinctoria* tuber. Water, ethanol, and methanol were used during the extraction process. High phenolic content was found in methanol extract (107.8 ± 0.025 µg/ml GAE) followed by water extract (92.4 ± 0.02 µg/ml GAE). High flavonoid content was also obtained in methanol extract (136.7 ± 0.04 µg/ml QE). Strong 2,2-diphenyl-1-picryl-hydrazyl (DPPH) scavenging activity was recorded for methanol extract with IC50 value of 44.4 µg/ml, compared with ethanol extract (97.54 µg/ml) and water extracts (98.24 µg/ml). The lower IC50 value of methanolic extract demonstrated strong antioxidant activity. The three elements that were most prevalent in *Impatiens tinctoria* tuber out of the eight elements examined were K (170 ± 0.05 mg/100 g sample), Ca (87 ± 0.08 mg/100 g sample), and Mg (16 ± 0.01 mg/100 g sample). The phenolics, flavonoids, and minerals found in *Impatiens tinctoria* A. Rich (Ensosila) tuber may protect against oxidative stress-related skin damage and thus deserving attention for future applications in cosmetics formulations.

## Introduction

*Impatiens tinctoria* A.Rich belongs to the family of *Balsaminaceae*^1^, generally known by its vernacular names, “Balsamine” in English and “Ensosila” in Amharic^2^. It grows in damp, shady localities in upland rainy forests, forest fringes and gullins, along streams and shady banks. In southern Tigray, Ethiopia, the plant has been cultivated for its tubers as a cash crop^3^. *Impatiens tinctoria* tuber has been utilized as traditional folk medicine for different diseases in Ethiopia. Its stem is chewed and used to treat fungal infections, mouth and throat disease, and for the aseptic cleaning of wounds. Its root decoction is drunk against abdominal pains and as purgative^2,4^.

Recent research revealed that the tuber has antifungal and antibacterial properties, demonstrating its varied medical uses^5^. Moreover, rural Ethiopian women frequently utilize *Impatiens tinctoria* tuber dye to color their hair, nails, and create detailed designs on their hands, palms, faces, and feet^6^. Its tuber is washed, pilled, sliced, steeped for at least 12 h, heated, mashed, and applied as paste for coloring hair, nails, and beard^2^. Red ink is also produced from ponded tuber juice. The tuber plays a significant function as traditional cosmetics because it is known to toughen the skin and improve skin health. However, it remains unknown whether Ensosila tuber is rich in antioxidants, flavonoids, phenolic compounds and minerals essential for skin health.

The traditional use of *Impatiens tinctoria* tuber for hand toughening is indicative of the fact that they are rich in anti-aging bioactive compounds. Recent studies also indicated that *Impatiens tinctoria* tuber is rich source of diverse secondary metabolites^2^. These compounds are responsible for the antimicrobial and wound healing activities^5,6^. The principal coloring matter for hair, hands and nail in *Impatiens tinctoria* tuber is 2-methoxy-1, 4-napthoquinone, which is a vitamin K analogue^7,8^. This compound have similar characteristics with Lawson of henna, because both contain a 1,4-naphtoquinone ring. To date, usefulness of this plant has not been proven for cosmetic and pharmaceuticals production.

The macro and micro elements are crucial for biological processes and synthesis of bioactive substances. They may also affect a compound’s ability to behave as an antioxidant. Plants contain minerals like calcium, phosphorus, iron, magnesium, potassium, copper, and others that help to keep the acid–base balance in bodily tissues. They aid in the absorption of proteins, lipids, carbs, and vitamins. Some of these minerals such calcium, copper, iron, magnesium, phosphorus, selenium, zinc, potassium and sodium are recognized as essential for skin health. Moreover, they are known to form peptide complexes that function as both carrier and signal molecules^9,10^. For instance, the tripeptide-copper complex (GHK-Cu) is frequently utilized to promote tissue remodeling and wound healing, acting as an anti-aging agent. However, cosmoceutical potential and the essential elements present in Ensosila tuber is not yet investigated. Evaluation of their antioxidant activity and mineral content is important for wider applications in cosmetic industries. Therefore, in this study, total phenolic, flavonoid, antioxidant activity and mineral content were studied.

## Materials and methods

### Plant materials

**Figure 1:**
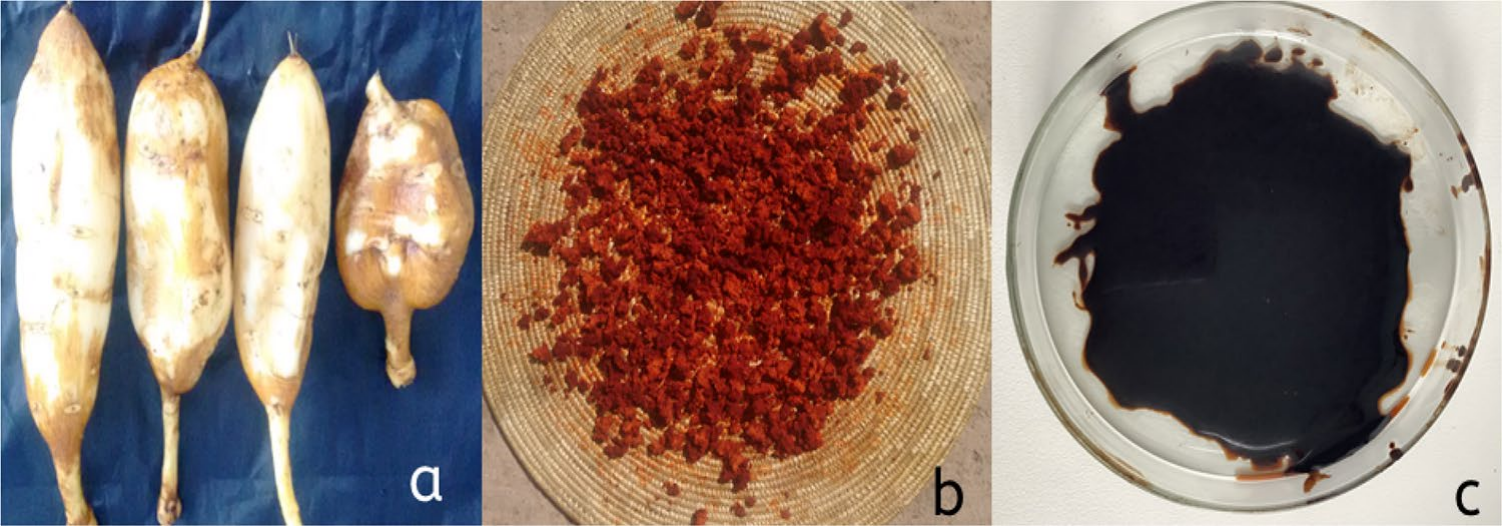
*Impatiens tinctoria* A. Rich used for extract preparation: (**a**) tuber (**b**) redish brown sliced tuber for air drying, (**c**) brown methanolic extract.

The vice President for Research and Technology Transfer at Addis Ababa Science and Technology University in Ethiopia granted academic permission for the study and collecting of the medicinal plant. The study complies with all relevant regulations and guidelines. The whole plant material of *Impatiens tinctoria* A. Rich was collected from Adama and Akaki local market, Ethiopia. To remove soil particles, the tubers were thoroughly washed in tap water and then rinsed with sterile distilled water (Fig. 1a). The tubers were divided into tiny pieces (Fig. 1b), air dried in the shade, then ground into a fine powder using a blender. The powder was weighed, sealed in polyethylene bags to avoid contaminants, and kept at room temperature for further extraction process. The plant material was presented to Botanist in AAU herbarium for identification and the voucher specimen GH-001 has been stored in Addis Ababa University (AAU) herbarium (ETH).

### Plant extract preparation

Tuber powder was mixed in a 1:20 ratio with extraction solvents (distilled water, ethanol, and methanol) to perform the phytochemicals extraction^2^. The extraction was carried out in a rotary shaker at 100 rpm for 24 h, filter by Whatman filter paper and concentrated by a rotary evaporator at 40 °C (Fig. 1c). The residual extracts were weighed and stored at 4 °C until use.

### Determination of total phenolic content

The Folin-Ciocalteu method was used to determine the total phenolic content. A 0.5 ml of extract was taken into 10 ml test tube containing 2.5 ml of a tenfold dilute Folin-Ciocalteu reagent and 2 ml of 7.5% sodium carbonate (Na_2_CO_3_). The mixture was vortexed, and allowed to stand for 30 min at room temperature in dark. After incubation at room temperature, the absorbance was measured at 765 nm against control, using UV–visible spectrophotometry. The calibration curve for gallic acid was made using concentrations of 0, 20, 50, 100, 150, 200, 250, 400, and 500 μg/ml of gallic acid as the standard. The total phenolic compound was expressed as μg gallic acid equivalents (GAE) per ml of plant extract^11^.

### Total flavonoid analysis

The flavonoid content of the *Impatiens tinctoria* tuber extract was determined using the aluminum chloride colorimetric technique. 0.5 ml of extract solution was taken into 10 ml test tube, containing 2.8 ml of distill water, 1.5 ml methanol, 0.1 ml of 10% aluminium chloride and 0.1 ml 1 M potassium acetate and were vortexed^12,13^. After vortexing at room temperature, the absorbance was measured at 415 nm using UV–visible spectrophotometry. The calibration curve for quercetin was made using concentrations of 0, 6.25, 12.5, 25, 50, 80, 100, 200, 400, and 500 μg/ml quercetin as the standard. The total flavonoid content was expressed as μg Quercetin equivalents (QE) per ml of the tuber extract.

### DPPH radical scavenging activity

The extract’s ability to scavenge DPPH radicals was assessed using 2,2-Diphenyl-1-picrylhydrazyl (DPPH). Three ml of the extract solution was taken and mixed with 1 ml of the DPPH solution (0.1 mM). After the mixture was allowed to stand for 30 min in dark at room temperature, absorbance was measured at 517 nm using UV–visible spectrophotometry. This approach was used to measured radical scavenging activity as the L-ascorbic acid equivalent, with L-ascorbic acid serving as the standard. L-ascorbic acid standard curve was produced for this purpose using concentrations of 0.062, 0.125, 0.25, 0.5, 1 and 2 M ascorbic acid. The extract scavenging activity was calculated as a percentage of the DPPH radical scavenged (I%), as shown in the equation below:

1 $$\left( {{\text{I }}\% } \right) \, = \left[ {\frac{(Absorbance\,\,\,of\,control - Absorbance\,\,\,of\,sample)}{{Absorbance\,\,of\,control}}} \right]*100$$

where absorbance of control is the absorbance of the control solution (containing all of the reagents, except the test sample^14^. The IC50 values were calculated by plotting DPPH percentage inhibition versus log transformed concentration of sample extract.

### Analysis of macro-minor elements

Mineralization of powdered *Impatiens tinctoria*(Ensosila) tuber (0.5 g) was performed in heat controlled microwave system with 9 ml 65% HNO_3_ in a closed vessel system having medium pressure rotor and heating in two stages (180 °C and power of 400 W for 15 min, 180 °C and power of 1200W for 5 min) followed by automatic cooling^15^. The digestate was then diluted to 5% NHO_3_ with deionized water. Particulates were removed by filtration through ashed glass fiber filter (0.45 μm) and stored at room temperature prior to analysis. Elemental determination was conducted in triplicates for seven of the nine mineral elements essential for skin health^16^. These include Na, Mg, Ca, K, Zn, Fe and Cu. The analysis was performed on ICP-MS. Ultrapure water was used for blanks. The results were expressed in mg/100 g dry weight of the sample using calibration standard according to ES3377:2014^17^.

### Statistical analysis

On the basis of triplicate data, the total phenolics, flavonoids, DPPH radical scavenging activity, and mineral content of the *Impatiens tinctoria* tuber were analyzed. To investigate this statistically, data were subjected to analysis of variance (ANOVA) using R version 4.3.1 and reported as mean and standard deviation of the mean (SD)^18^.

## Results and discussion

Due to their good biological activities and lesser side effects, cosmeceutical plants have attracted considerable attention in the global cosmetic industries. Vitamins, antioxidants, oils, dyes, and other bioactive compounds from plants are used in cosmetic formulations. These compounds are extracted from the leaves, flowers, stems, barks, tubers, and roots of plants. Only few of the cosmeceutical plants are exploited for commercial purpose, despite the existing worldwide plant biodiversity. *Impatiens tinctoria* (Ensosila) species are one of them, and they are important in supplying Ethiopia’s rural women with the cosmetics and medicines requirement. The present study indicated that *Impatiens tinctoria* (Ensosila) tuber had a high browning activity when fresh tuber sliced (Fig. 1b). Such browning characteristics are linked to antioxidant and phenolic activities. The browning property was also documented for *Impatiens tinctoria* A.Rich collected from Butajira area, Ethiopia by Degu et al.^2^. A similar browning property of *Dioscorea pentaphylla* tuber was also reported by Kumar et al.^19^.

### Total phenolic content of the extracts

Total phenolic content was determined from the calibration curve of gallic acid y = 0.005x + 0.038, R^2^ = 0.99, where y is absorbance at 765 nm and x is total phenolic content in the extracts of *Impatiens tinictoria*(Ensosila) expressed in μg/ml gallic acid equivalent. The total phenolic content is presented in Table 1 below. There was a significant difference in total phenolic content of the extracts (*p* < 0.05). The methanolic extract had the highest total phenolic content (107.8 ± 0.025 µg/ml), followed by the water extract (92.4 ± 0.02 µg/ml). On the other hand, ethanol extracts have relatively low phenolic content compared with methanol extract.

**Table 1:** Total phenolic and flavonoid content of the tuber extracts.

Extract	Phenolic content (μgGAE/ml) ± SD	Flavonoid content (μgQE/ml) ± SD
Water	92.4 ± 0.02^a^	117.17 ± 0.03^a^
Ethanol	78.86 ± 0.04^b^	100 ± 0.003^b^
Methanol	107.8 ± 0.025^c^	136.7 ± 0.04^c^

*GAE* gallic acid equivalent, *QE* quercetin equivalent, Difference in means indicated by different letters(^a,b,c^) in the same column which are significantly different at *p* = 0.05.

Recovery of phenolic compounds during extraction is inhibited by the polarity of the extraction solvent and the compound’s solubility^13,20^. Amongst the various solvents, there were significant differences in the total phenolic compounds recovered from *Impatiens tinctoria* tuber. When compared to other solvent systems, it was found that methanol and water were the most effective solvents for extracting phenolic compounds from *Impatiens tinctoria* tuber. Due to their positive functions as antioxidants, antimicrobials, antimutagens, and for the repair of damage caused by oxidative stress, plant phenolic compounds; have attracted significant attention in the food and pharmaceutical industries^8,21^. A number of human diseases, including cancer and aging, have been linked to oxidative stressors. The hydroxyl groups of phenolic compounds provide them the capacity to scavenge free radicals. The presence of high phenolic content in methanol extracts (107.8 μg GAE/ml) of *Impatiens tinctoria* may make it a promising antioxidant to help body fight microbial infection and aging. Phenolic content of 7684 μgGAE/ml for Jerusalem artichoke tuber extract^22^, 19.2 mg/g for *Dioscorea pentaphylla*^19^, and 2400 mgGAE/kg for *Colocasia esculenta*^23^ were reported, which shows variation from species to species, extraction and analysis protocols used. Antimicrobial activity of *Impatiens tinctoria* A Rich tuber extract have been previously reported by Degu et al.^2^. However, further study will be needed to identify the bioactive phenols.

### Total flavonoid content of the extracts

Total flavonoid content was determined from the calibration curve of quercetin y = 0.0015X + 0.62, R^2^ = 0.99, where y is absorbance at 415 nm and x is total flavonoid content in the extracts of *Impatiens tinctoria* expressed in μg/ml quercetin equivalent. In Table 1, the results for total flavonoid content is presented. Nearly all solvent extracts have significant flavonoid content. Methanol extract has the highest flavonoid concentration (136.7 ± 0.04 μg QE/ml), followed by water (117.17 ± 0.03 μg QE/ml) and ethanol extracts (100 ± 0.003 QE/ml), respectively (Table 1). Earlier study by Degu et al.^2^ on *Impatiens tinctoria* tuber extract for flavonoids using general test also indicated their presence. Total flavonoid contents of 11.2 mg/g for *Dioscorea pentaphylla*^19^, 605 mg/ml for Jerusalem artichoke tuber extract^22^, 2050mgGAE/kg for *Colocasia esculenta*^23^ were reported, which shows variation from species to species, extraction and analysis protocols.

Plant-derived flavonoids are non-nutrient components that have been shown to improve cells’ resistance to oxidative stress. They have a planar structure, free hydroxyl groups that vary in number and position, and a C2–C3 double bond. These substances are crucial for the chelation of metals, scavenging of free radicals, stabilization of scavenging chemicals, and inhibitions of enzymes that produce free radicals^24^. The presence of high flavonoid in *Impatiens tinctoria* extracts indicates its potential as an antioxidant. However, further studies on the flavonoid constituents and their antioxidant mechanisms are required.

### DPPH scavenging activity

**Figure 2:**
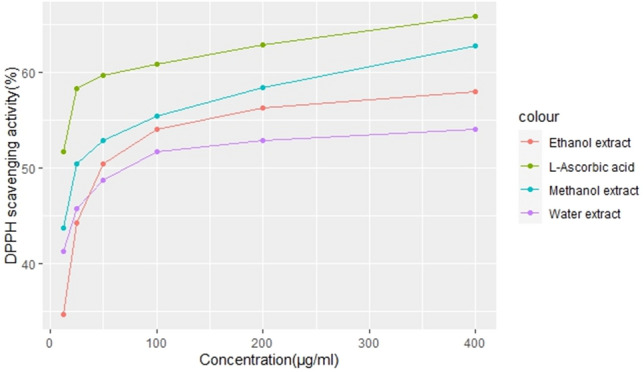
Concentration dependent DPPH scavenging activity of the *Impatiens tinctoria* A.Rich tuber extract.

**Figure 3:**
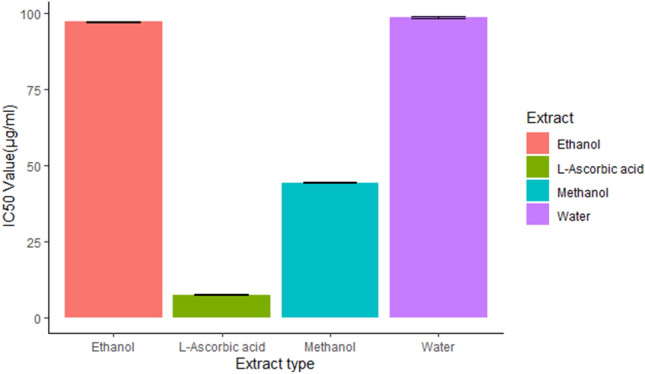
IC50 value of the *Impatiens tinctoria* A.Rich (Ensosila) tuber extracts, bar and error bars indicate the mean value and standard deviation of replicated data, respectively.

In terms of DPPH scavenging activity, *Impatiens tinctoria* A.Rich tuber extract demonstrated a concentration response relationship. Increased scavenging ability is correlated with increased extract concentration. The DPPH scavenging activity of the extracts ranged from 34.7 to 62.8%, in ethanol and methanol extracts, respectively. In comparison to ascorbic acid (positive control), methanol extracts showed the highest percent DPPH scavenging activity (62.8%) at highest tested extract concentration of 400 µg/ml (Fig. 2). Ethanol and water extracts showed 58.4% and 54.1% DPPH scavenging activity, respectively, at 400 µg/ml extract concentration. At lower concentration of the tested extract (12.5 µg/ml), more than 50% of the antioxidant activity was exhibited for all of the extracts. The IC50 value of Methanol extract is twofold lower (44.4 µg/ml) than ethanol (97.5 µg/ml) and water extract (98.2 µg/ml (Fig. 3)).

The DPPH method is frequently used to assess the antioxidants’ capacity to scavenge free radicals^14^. DPPH (2, 2- diphenyl-1-picrylhydrazyl) is a very stable organic free radical and presents the ability of accepting an electron or hydrogen radical. Antioxidants interact with DPPH to transfer their hydrogen or electron, which results in the DPPH solution becoming discolored. Stochiometrically, the amount of electrons that are taken up determines how much the solution is bleached^24^. The decrease in its absorbance of the extracts at 517 nm indicates the antioxidants reduction capacity of DPPH radical^14,25^. In this experiment, highest antioxidant activity (62.8%) was observed at 400 µg/ml methanol extract. Lubag et al.^26^ reported antioxidant activity of 61% using lipid peroxidation for *Satyrium nepalenses* tuber methanol extract. More than 71% of antioxidant activity was also reported for tuber extracts of *Cyclamen mirabile* Hildebr using thiocyanate method^27^. Besides, more than 50% of the antioxidant activity was maintained at lowest extract concentration tested for all the extracts. Similar result was reported by Kumar et al.^19^ from *Dioscorea pentaphylla* tuber extract, where low concentration of the tuber extract tested maintained 50% of the antioxidant activity. This experimental result indicates that the methanol extract of *Impatiens tinctoria* tuber has antioxidant activity compared with the standard. To the best of my knowledge, this is the first report on the antioxidant potential of *Impatiens tinctoria* A. Rich (Ensosila) tuber extract.

The solvent system, plant tissue, genotype and plant species difference used also affected the extracts DPPH scavenging activity (Fig. 2). The low IC50 value of methanol extract indicates strong scavenging capacity produced by the phenolic compounds present in the methanol extracts. Mishra et al.^28^ reported IC50 value (30.79 µg/ml) using DPPH assay for *Satyrium nepalenses* tuber methanol extract, which is lower than the experimental result presented here. A closer IC50 value of 49.17 ± 1.26 µg/ml was also reported from leaf methanolic extract of *Lippia adoensis* (koseret; Ethiopian spice)^29^. However, less scavenging IC50 value of 82.07 µg/ml was reported for *Dioscorea pentaphylla* tuber methanol extract^19^. The result indicates that 50% antioxidant activity was exhibited at lowest extract concentration tested (12.5 µg/ml). Niziol-Lukaszewska et al.^22^ also documented similar result for Jerusalem artichoke tuber extract.

However, ethanol and water extracts had twofold higher IC50 value compared with methanol and L-ascorbic acid, indicating less scavenging power. The findings indicated that methanol is effective extraction solvents with significant DPPH radical scavenging activity.

### Mineral elements analysis

Potassium is the most abundant of the major essential elements in the *Impatiens tinctoria* A.Rich tuber, followed by Ca, Mg, and Na. Among the macroelements, K (170 ± 0.05 mg/100 g) and Ca (87 ± 0.08 mg/100 g) were in the highest amount. Minor elements of special importance for skin health were also detected in *Impatiens tinctoria* A.Rich tuber powder. Among the minor elements, Fe (3.4 ± 0.03 mg/100 g) was in the highest content followed by Zn (2.6 ± 0.12 mg/100 g) and Cu (0.4 ± 0.24 mg/100 g) (Table 2). High mount K, Mg, Ca and sodium were also reported for *Colocasia esculenta* tuber^30^, *Kedrostis Africana* (L) Cogn tuber^31^, *Dioscorea dumetorum* Pax tuber^32^, which were in agreement with this experimental result.

**Table 2:** The macro-minor elements composition of *Impatiens tinctoria* A.Rich tuber.

S. no.	Element analyzed	Content, in mg/100 g dry weight of the sample (mean ± SD)
1	Sodium	9.2 ± 0.03
2	Magnesium	16 ± 0.01
3	Potassium	170 ± 0.05
4	Calcium	87 ± 0.08
5	Iron	3.4 ± 0.03
6	Zinc	2.6 ± 0.12
7	Copper	0.4 ± 0.24

Literature reports indicated nine crucial minerals for skin health. Among them, calcium, copper, iron, magnesium, phosphorus, selenium, zinc, potassium, and sodium are included in the list^16^. Moreover, the mineral makeup of a plant extract can affect its antioxidant effectiveness. Certain metals behave as pro-oxidants^33^. K, Ca, Mg, and Na are the essential major elements found in *Impatiens tinctoria* A.Rich tubers. It is well recognized that magnesium and calcium play a part in keratinocyte proliferation, differentiation, and wound healing. Fe, Zn, and Cu are among the minor essential elements detected in *Impatiens tinctoria* tuber. Fe is the cofactor for various oxygenase enzymes. Zinc is a skin protectant and is frequently used as a wound healing agent. Zn is occasionally regarded as an unconventional antioxidant^16^. Tyrosinase, an enzyme involved in skin pigmentation, collagen crosslinking, and healthy hair development, requires copper as a cofactor. The presence of major and minor elements essential for skin health demonstrates potential of *Impatiens tinctoria* tuber for anti-aging skin care products development.

## Conclusion

This study showed that the extracts of *Impatiens tinctoria* A.Rich tubers contain significant amounts of phenolic and flavonoid compounds. Also, its DPPH scavenging activity indicated that the tuber is a substantial source of physiologically active compounds and has significant antioxidant activity. Due to their high polyphenol content, methanol extracts demonstrated remarkable free radical scavenging activity. Moreover, the tuber provided a good source of macro and microelements crucial for skin health, with potassium, calcium, and magnesium being the most prevalent. The findings of this result and its substantial history of use for cosmetic purpose support the potential of the medicinal plant for cosmetic formulation. For better understanding, isolation and structure elucidation of individual phenolic, flavonoid compounds, and 2-methoxy-1, 4-napthoquinone and their in vitro and in vivo studies are recommended.

## Data Availability

The datasets utilized and analyzed during this investigation are available upon reasonable request from the author.
